# Nicotine-Cadmium Interaction Alters Exploratory Motor Function and Increased Anxiety in Adult Male Mice

**DOI:** 10.1155/2014/359436

**Published:** 2014-11-12

**Authors:** Duyilemi Chris Ajonijebu, Philip Adeyemi Adeniyi, Adeshina Oloruntoba Adekeye, Babawale Peter Olatunji, Azeez Olakunle Ishola, Olalekan Michael Ogundele

**Affiliations:** ^1^Department of Physiology, College of Medicine and Health Sciences, Afe Babalola University, Ado-Ekiti, Ekiti State, Nigeria; ^2^Department of Anatomy, Cell Biology and Neuroscience Unit, College of Medicine and Health Sciences, Afe Babalola University, College Building II, Room G14, Km 8.5 Afe Babalola Way, PMB 5454, Ado-Ekiti, Ekiti State, Nigeria; ^3^Department of Biological Sciences, College of Sciences, Afe Babalola University, Ado-Ekiti, Nigeria; ^4^Department of Anatomy, College of Health Sciences, University of Ilorin, Ilorin, Kwara State, Nigeria

## Abstract

In this study we evaluated the time dependence in cadmium-nicotine interaction and its effect on motor function, anxiety linked behavioural changes, serum electrolytes, and weight after acute and chronic treatment in adult male mice. Animals were separated randomly into four groups of *n* = 6 animals each. Treatment was done with nicotine, cadmium, or nicotine-cadmium for 21 days. A fourth group received normal saline for the same duration (control). Average weight was determined at 7-day interval for the acute (D1-D7) and chronic (D7-D21) treatment phases. Similarly, the behavioural tests for exploratory motor function (open field test) and anxiety were evaluated. Serum electrolytes were measured after the chronic phase. Nicotine, cadmium, and nicotine-cadmium treatments caused no significant change in body weight after the acute phase while cadmium-nicotine and cadmium caused a decline in weight after the chronic phase. This suggests the role of cadmium in the weight loss observed in tobacco smoke users. Both nicotine and cadmium raised serum Ca^2+^ concentration and had no significant effect on K^+^ ion when compared with the control. In addition, nicotine-cadmium treatment increased bioaccumulation of Cd^2+^ in the serum which corresponded to a decrease in body weight, motor function, and an increase in anxiety.

## 1. Introduction

Wide arrays of behavioural and physiological responses have reportedly been observed following nicotine use either in tobacco smoke or as a pure drug [1]. In human abusers and rodent models, weight loss, anxiety, depression, and motor dysfunctions are among the most frequently reported behavioural changes [2–4]. Long and short term use of nicotine in tobacco smoke alter synaptic function, neurotransmitter level, and the anatomical structure of the brain to varying extents. Similarly, the addictive effect of nicotine in tobacco smoke is known to potently alter the expression of cholinergic receptors, serum level of ions, and neurotransmission (central and peripheral) [5]. Primarily, nicotine, being a structural analogue of acetylcholine, potentiates the receptors and facilitates the increase in intracellular Ca^2+^ concentration, vesicle formation, and neurotransmission at synapses and neuromuscular junctions. However, prolonged excitation of cholinergic synapses by nicotine leads to excitotoxicity and synaptic dysfunction, observed in prolonged use and abuse of nicotine [6–11].

Although nicotine gets into the body through various means—such as chewing of tobacco leaves and absorption of nicotine through the skin [12, 13]—the most predominant route of nicotine use is through tobacco smoke in cigarettes and pipes (respiratory) [14, 15]. Interestingly, several studies have reported the presence of cadmium, carbon monoxide, lead, cooper, silicon, and alkaloids in the tobacco smoke [14, 16]. Despite the striking evidence of the bioaccumulation of cadmium in tobacco smoke, coupled with its ability to inhibit cellular respiration in neurons, the metabolic and behavioural changes associated tobacco smoke has often been restricted to nicotine and its associated functions. Although cadmium is a trace element in the blood, previous studies have shown that the level of cadmium in the serum of smokers is usually 30–50% higher than that of nonsmokers [17–19]. Furthermore, overproduction of carbon dioxide, due to cadmium-mediated mitochondria stress, has been reported to induce synaptic inhibition at cholinergic endings in the nervous system [18, 19]. Consequently, the reactive oxygen species (ROS), generated from cadmium blockade of* cytochrome a3* and* cytochrome C*, participates in the elevation of cellular calcium concentration, peroxidation of neuronal membrane, and degeneration of neurons [20–23].

The bioavailability and effect of nicotine and (or) cadmium in the blood are known to be dependent on the duration of exposure to these substances either solely or combined in tobacco smoke [24–26]. Similarly, differences in the magnitude of addiction or degenerative changes seen in the brain, after a time-dependent tobacco smoke abuse, vary with the sex, age, and the developmental stage of the individual [27, 28]. Females, generally, are more sensitive to nicotine addiction than males, which also show less toxicity compared with the females [27]. Consequently, the long term effect of tobacco abuse in males has been found to be linked with irrational behaviour, violence, anxiety, mood disorders, movement disorders, and weight loss [27, 29–31]. Furthermore, movement and anxiety are affected due the effect of nicotine on reward brain regions and brain stress systems involving dopaminergic regulation, epinephrine, neuropeptide Y, and neuromuscular nicotinic stimulation [31–34].

Although the effect of nicotine (tobacco) on metabolism, anxiety, motor function, and brain reward has been studied extensively, the specific role of cadmium-nicotine treatment on these parameters (metabolism, anxiety and motor function) are yet to be elucidated, comparatively, the effect of cadmium, nicotine, and a combination of nicotine-cadmium treatment in male adult mice. We investigated pharmacologically the effect of nicotine and (or) cadmium treatment on the body weight, motor function, and anxiety linked behavioural changes during acute and chronic treatment phases. Furthermore, we sought to identify the synergistic effect of nicotine and cadmium on metabolism, anxiety, and exploratory motor dysfunction seen in tobacco abuse.

## 2. Materials and Methods

Cadmium chloride and nicotine were a generous gift form Dr. Maria Deluca of the University of Cagliari, while the normal saline used for the preparation was procured from Kanada Pharmaceuticals, Nigeria. The test chemicals were prepared weekly in normal saline and applied within the same period.

### 2.1. Treatment

Male adult albino Swiss mice (*N* = 24), each weighing between 19 and 22 gms, were used for this study. The animals were obtained from the animal holding facility of Obafemi Awolowo University, Nigeria, and allowed to acclimatize for 7 days before the commencement of treatment. Subsequently, four (4) separate groups of *n* = 6 animals each were created at random and maintained in a standard laboratory environment with controlled humidity, pressure, and temperature. Treatment protocols were in accordance with the ethical requirements of the Animal Use and Care Committee of the Afe Babalola University, Nigeria. Animals (all groups) were treated once daily for a duration of 21 days (D1–D21); days 1–7 represent the acute treatment while days 7–21 are the chronic treatment phase. A group of *n* = 6 mice received 1.4 mg/Kg BW of cadmium chloride prepared in normal saline (intramuscular). Another group received 0.4 mg/Kg BW of nicotine (subcutaneous) [35, 36] while a combined treatment group received nicotine (subcutaneous; 0.4 mg/Kg) and cadmium (intramuscular 1.4 mg/Kg) [37, 38]. The control was treated with normal saline (subcutaneous) for the duration of the experiment (Table S1 in the Supplementary Material available online at http://dx.doi.org/10.1155/2014/359436).

### 2.2. Weight Measurement

Average weight per group (gms) was measured at days 1, 7, 14, and 21 using a sensitive weighing balance (Jenway) and plotted (ANOVA) to compare weight changes for the treatment groups versus the control.

### 2.3. Open Field Test

This was done to determine exploratory motor function relative to anxiety linked behavior in a single test [39]. The animals were trained in the open field area (OFA) continually for 4 days before the actual test to facilitate acclimatization to the test equipment and environment. In addition, the animals were assisted to move or navigate the open field area when necessary during the training phase. OFA was made of white wood with dimensions, 100 cm wide, 100 cm long, and 45 cm high, and was marked with dark lines dividing the floor into 16 uniform squares and a center square. A high definition video recording system (SONY) was placed in position to capture all corners and sides of the OFA in order to record animal movement and behavior. For the actual test, animals were placed in the OFA and allowed to explore for 5 minutes following which analysis was done for motor and anxiety related functions.

### 2.4. Evaluation of Motor Function

This was done by estimating the average number of lines crossed per animal/per group for the duration of the test (5 minutes) and was expressed as the frequency of lines crossed after the acute and chronic treatment phases.

### 2.5. Anxiety Linked Behaviors

This was evaluated in relation to the observed motor functions in the OFA. Center square duration, frequency of grooming, and rearing were estimated and analyzed after the acute and chronic treatment phases to determine the level of anxiety linked with exploratory motor behavior.

### 2.6. Blood Electrolyte Concentration

At the end of the chronic phase, the animals were euthanized humanely to obtain blood through cardiac puncture. Subsequent processing involved the use of anticoagulant (heparin), followed by centrifugation at 4°C to obtain the cold serum. The concentrations of Ca^2+^, K^+^, Na^+^, and Cd^2+^ were determined in a mass atomic spectrometry as previously described [40]. Serum was diluted and spiked with enriched radioisotopes following which the primary standards were measured in analogue mode for *n* = 6 samples per group.

### 2.7. Statistical Analysis

Data was plotted in ANOVA for all behavioral tests, weight, and electrolyte concentrations from mass spectrometry. Statistical significance was determined using GraphPad Prism (Version 6) (^*^ *P* < 0.05) and expressed as mean ± SEM (standard error of mean).

## 3. Results

### 3.1. Change in Average Weight

**Figure 1:**
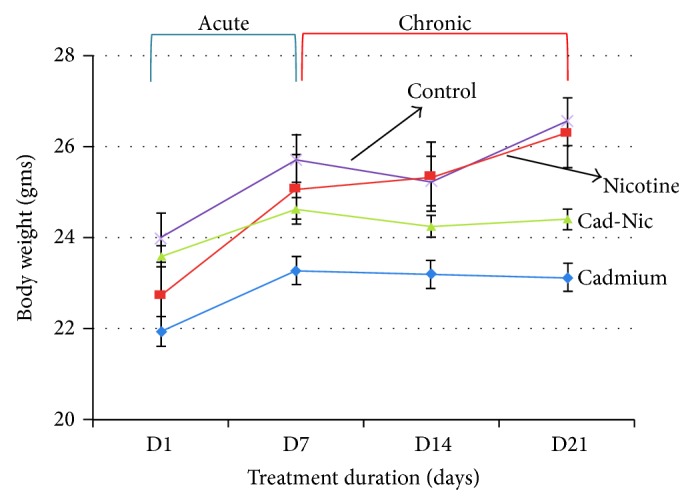
Comparative body weight analysis for Cd, Nc, Cd-Nc, and control group after the acute and chronic treatment phases. As a result of acute nicotine treatment, an increase in body weight was observed in the acute (D1–D7) and chronic treatment phases (D7–D21). However, comparing the nicotine treated animals and the control, only a slight change in body weight was seen (*P* < 0.05). Administration of cadmium caused an increase in body weight after the acute phase; subsequently, a decline in body weight was seen after chronic treatment with cadmium (*P* < 0.001) versus the control. Similarly, combined treatment with nicotine and cadmium induced an increase in body weight after the acute treatment phase, followed by a decline after chronic treatment. In addition, the decrease in body weight observed in the nicotine-cadmium treatment group was lower than the average body weight for nicotine treatment but higher than the weight recorded in cadmium treatment group. The average weight/ group was expressed as mean ± standard error of mean (SEM) (^*^ *P* < 0.001, Cd^2+^ versus Nic; ^∧^ *P* > 0.05, Cd^2+^ versus Cd^2+^ + Nic; ^×^ *P* > 0.05, Nic versus Cd^2+^ + Nic; ^#^ *P* < 0.001, Cd^2+^ versus control; ^§^ *P* > 0.05, Nic versus control; and ^*α*^ *P* > 0.05, Cd^2+^ + Nic versus control).

The comparative weight analysis for nicotine, cadmium, or a combined treatments showed that nicotine treatment caused a significant increase in body weight (*P* < 0.05) while cadmium (*P* < 0.001) treatment induced a decline in body weight when compared with the control. Similarly, a combined nicotine-cadmium treatment decreased the body weight of the animals versus the control (*P* < 0.01) during the acute phase (D1–D7). However, following chronic administration (D7–D21) further decline in weight was observed in the cadmium and cadmium-nicotine treatment groups when compared with the control (*P* < 0.01). Nicotine treatment caused a significant increase in average body weight during the chronic treatment phase recording a significance of *P* < 0.05 versus the control (Figure 1). These findings suggest that the weight loss associated with tobacco smoke is a result of cadmium or tobacco cadmium synergy rather than nicotine only; nicotine treatment increased the body weight both in the chronic and acute phases.

### 3.2. Metal Ion Analysis in Serum

#### 3.2.1. Sodium (Na^+^)

**Figure 2:**
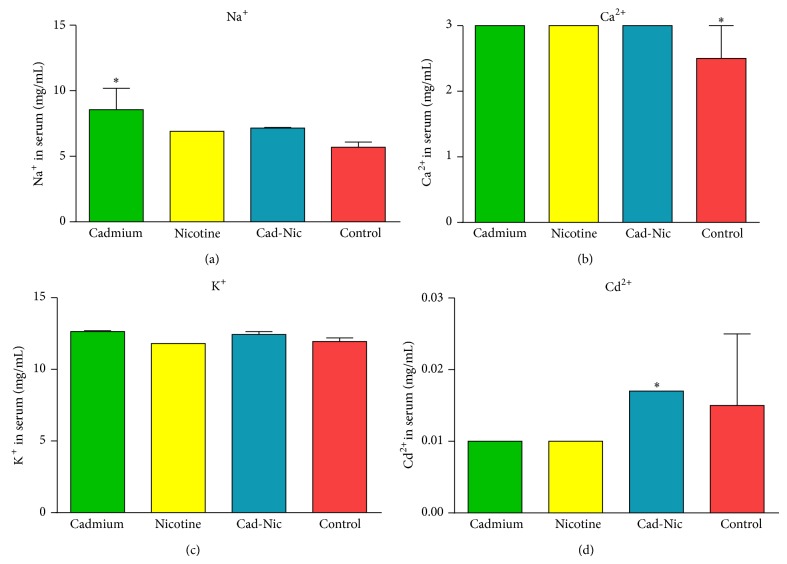
Concentration (mg/dL) of Na^+^, K^+^, and Ca^2+^ and cadmium in the blood. (a) Bar chart showing the level for Na^+^ in blood (mg/mL). An increase in Na^+^ was associated with cadmium treatment when compared with the control (*P* < 0.05). (b) An empirical increase in Ca^2+^ concentration was observed in the blood of the treatment groups when compared with the control (*P* < 0.05). (c) Concentration of K^+^ in blood; no significant change was seen in the K^+^ ion concentration after nicotine and (or) cadmium treatment. (d) Bar chart demonstrating the blood level of cadmium in the treatment groups and control. An increase in Cd^2+^ was observed in the nicotine-cadmium treatment versus the control (*P* < 0.05).

Spectrum analysis showed that cadmium treatment increased serum Na^+^ ion level significantly above the values observed for the control. No significant change was observed in the nicotine and nicotine-cadmium treatment group when compared with the control (Figure 2(a)).

#### 3.2.2. Calcium (Ca^2+^)

The concentration of calcium in the serum of the treatment groups showed no significant change among the treatment groups for the duration of treatment (21 days). The control group recorded the lowest calcium concentration in the blood when compared with the treatments. This suggests the role of calcium in the activity of nicotine and cadmium and the significance of raised calcium levels in both cadmium and nicotine toxicity (Figure 2(b)).

#### 3.2.3. Potassium (K^+^)

Although alterations in potassium levels were statistically insignificant, empirically, cadmium treatment and nicotine-cadmium treatment groups showed an increase in K^+^ ion level when compared with the nicotine treatment and the control. The potassium ion concentration change also correlated with Ca^2+^ ion level change in the treatment and control groups (Figure 2(c)).

#### 3.2.4. Cadmium (Cd^2+^)

The cadmium concentration in the cadmium and nicotine treatments reduced significantly when compared with the control and the nicotine-cadmium treatment groups. In the cadmium-nicotine treatment, blood Cd^2+^ level increased significantly when compared with the control, cadmium, and nicotine treatment groups (Figure 2(d)). This suggests the role of nicotine in increased bioaccumulation of Cd^2+^ in the blood of tobacco smokes. Also, we observed that cadmium treatment was associated with reduced serum Cd^2+^; although the mechanism involved is unclear, it is suspected to have resulted from the rapid clearance from the blood in urine and other physiological means (positive feedback).

### 3.3. Motor Function

**Figure 3:**
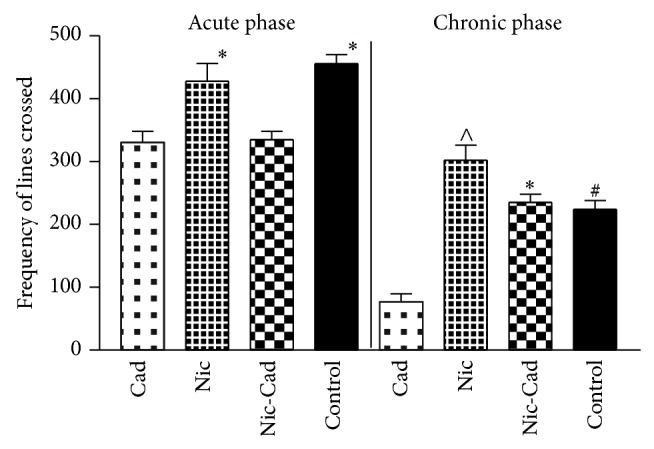
Motor function analysis in open field test (OFT) for Cd^2+^ and nicotine toxicity. Bar chart (acute phase) representing the frequency of lines crossed in OFT for the treatment groups and control after the acute (D1–D7) and chronic treatment phases (D7–D21). No significant change in motor function was observed between the treatment groups and control after the acute phase. However, after the chronic phase, the cadmium (*P* < 0.01) treated group showed a decline in motor function while the nicotine treatment showed an increase in exploratory motor function when compared with the control (*P* < 0.05). Similarly, the nicotine-cadmium treatment recorded a significant increase in motor function when compared with the cadmium treatment group (*P* < 0.05) and control.

The open field test (OFT) was employed to evaluate exploratory motor functions after the acute phase (D1–D7) and the chronic phase (D7–D21). Subsequent analysis involved determination of the frequency of lines crossed in the open field area (OFA) for the nicotine, cadmium, nicotine-cadmium, and control groups. After the acute treatment phase, nicotine treatment caused an increase in exploratory motor activity when compared with the cadmium and nicotine-cadmium treatment groups (*P* < 0.05). Similarly, the cadmium and nicotine-cadmium treatment recorded a significant decline in motor function versus the control (*P* < 0.01). After chronic treatment (D21), the nicotine group recorded an increase in motor function when compared with the control (*P* < 0.05). Also, the nicotine-cadmium treatment showed an increase in motor function versus the control (*P* < 0.05) while cadmium treatment recorded a significant decline in exploratory motor function versus the control (*P* < 0.001) (Figure 3).

### 3.4. Centre Square Duration (CSD)

**Figure 4:**
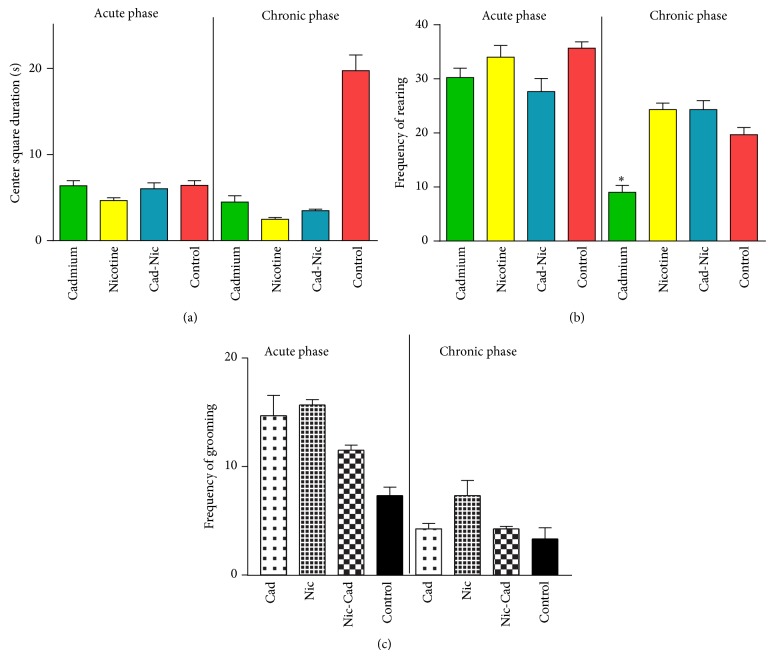
(a) Anxiety related behavioral analysis (I) in open field test (OFT) for Cd^2+^ and nicotine toxicity. Bar chart representing the center square duration (CSD) in OFT for the treated groups and control after the acute (D1–D7) and chronic (D7–D21) treatment phases. The duration spent in the center square of the open field area was estimated to determine the mean duration for each group at the end of the acute and chronic phases. After the acute treatment phase, no significant change in anxiety linked behavior was seen in the nicotine and (or) cadmium treatment groups. After chronic treatment, cadmium and nicotine treatment increased anxiety linked behavior in the animals, as shown by a significant decrease in CSD (D7–D21). Similarly, a combined nicotine-cadmium treatment also caused an increase in anxiety linked behavior (reduced CSD) when compared with the control (^∧^ *P* < 0.001). (b) Anxiety related behavioral analysis (II) in open field test (OFT) for the treatment and control groups. The mean frequency of rearing was determined for the nicotine and (or) treatment groups versus the control after the acute (D1–D7) and chronic (D7–D21) treatment phases. No significant change in the frequency of rearing for the treatment groups after the acute treatment phase. After the chronic phase, subsequent tests show a significant reduction in rearing time (↑anxiety) for the cadmium treatment group when compared with the control, nicotine, and nicotine-cadmium treatment groups (^***^ *P* < 0.001). (c) Anxiety related behavioral analysis (III) in open field test (OFT) for Cd^2+^ and (or) nicotine toxicity. Bar chart representing the frequency of grooming in OFT for the treatment groups and control after the acute (D1–D7) and chronic (D7–D21) treatment phases. No significant change in the frequency of grooming was observed after the acute treatment phase. Similarly, no significant change was seen in the frequency of grooming after the chronic treatment phase for all treatment groups versus the control.

No significant change was observed in the CSD after the acute treatment phase (Figure 4(a)) when the treatment groups were compared with the control. However, after the chronic treatment phase, nicotine, cadmium, and nicotine-cadmium treatment showed a significant decrease in CSD (*P* < 0.001) when compared with the control. This was observed as an increase in time spent exploring the corners and walls of the open field area (↑anxiety) (Figure 4(a)).

### 3.5. Frequency of Rearing

No significant change was seen after the chronic phase when the treatment groups were compared with the control. Similarly, the cadmium treatment group showed a decrease in the frequency of rearing versus the control (*P* < 0.001). This was similar to our observations in frequency of lines crossed (Figure 3) and the CSD (Figure 4(a)). Generally, the cadmium treatment group showed a decline in motor function and an increase in anxiety related behaviour when compared with the nicotine or nicotine-cadmium treatment (Figure 4(b)).

### 3.6. Frequency of Grooming

No significant change was observed in the treatment groups after the chronic and acute phase versus the control (Figure 4(c)).

### 3.7. Analysis of Anxiety in OFT

From these findings, we deduced that a time-dependent relationship exists between the use of nicotine and (or) cadmium and the observed increase in anxiety or decreased motor function. The most significant decline in exploratory motor function and increase in anxiety were observed after chronic treatment of cadmium while no significance was recorded after acute treatment. In addition, nicotine increased motor function and anxiety after the acute and chronic phases when compared with the control. A combination of nicotine and cadmium reduced the exploratory motor activity and increased anxiety in the animals after the chronic phase, thus suggesting the complimentary role of cadmium in observed anxiety related behavioural changes seen in prolonged tobacco use (Figures 4(a)–4(c)).

## 4. Discussion

Taken together, the outcomes of this study prove that cadmium is involved in the weight, anxiety, and motor changes observed in tobacco (nicotine) use. We observed that nicotine treatment increased the body weight after the acute and chronic treatment phases while cadmium caused a decrease in weight versus the control. Interestingly, nicotine-cadmium treatment, similar to tobacco use, caused a decline in body weight after the chronic treatment phase. Subsequent analysis reveals that an increase in exploratory motor activity was associated with nicotine use after the acute and chronic treatment phases; however, an increase in anxiety was also observed in this group. Consequently, cadmium and nicotine-cadmium treatment induced a decline in exploratory motor activity and increased anxiety after the chronic phase, further suggesting the complimentary effect of cadmium on nicotine induced anxiety and decreased motor coordination observed in chronic tobacco smoke exposure.

Furthermore, the effect of cadmium and nicotine was also examined on electrolyte balance and bioaccumulation of cadmium in the serum after the chronic phase (D21). We observed that chronic cadmium treatment increased serum Ca^2+^ and Na^+^ ion concentration when compared with the control (Figures 2(a) and 2(b)). Similarly, nicotine increased serum Ca^2+^ ion level, while no changes in K^+^ and Cd^2+^ ion concentration were observed in nicotine and cadmium treatment groups (Figures 2(c) and 2(d)). Combined nicotine-cadmium treatment did not cause any significant change in serum Ca^2+^, Na^+^, and K^+^ ion concentrations when compared with the control (Figures 2(a)–2(c)). Interestingly, the nicotine-cadmium treatment increased the serum level of Cd^2+^ ion significantly versus the control, nicotine, and cadmium treatment groups (Figure 2(d)); this suggests the role of nicotine in bioaccumulation of cadmium observed in chronic exposure to tobacco smoke. The observed effects of nicotine, cadmium, and nicotine-cadmium combination suggest a rapid change in metabolism which is dependent on the dose and duration of exposure [41, 42]. Furthermore, nicotine and cadmium as separate entities have been implicated in neurotoxicity and cell death by altering energy metabolism, signal transduction, reward, and induction of brain stress [43–46]. Previous studies have shown that the anxiety and exploratory motor changes associated with acute and chronic tobacco use involved alteration in neurotransmitters such as GABA [47, 48], dopamine [49, 50], and norepinephrine in brain [51]. Similarly, the nicotine in tobacco smoke affects the level of acetylcholine and epinephrine in the neuromuscular junction, arteries, glands, and other viscera [51, 52].

Dopaminergic D_1_ and D_2_ stimulation in the hippocampus has been implicated in nicotine-mediated anxiety and reward system [46–48, 53]. Nicotinic modulation of dopaminergic system is believed to be involved in increased motor function, brain reward, addiction, anxiety, and late onset Parkinson's disease observed in smokers [54, 55]. However, nicotinic stimulation of acetylcholine receptors induces the release of acetylcholine and norepinephrine in the central and peripheral nervous system and has been found to involve transient calcium release and release of high frequency pulses associated with anxiety and motor dysfunctions [56]. Interestingly, cadmium and the associated carbon dioxide produced in oxidative stress have been linked with the inhibition of synaptic activity at cholinergic endings [57, 58]. Although, its actual role in the modulation of neurotransmitter and reward system is elusive, our findings suggest nicotine increases the serum concentration of cadmium when both molecules are coadministered. As a result, both nicotinic stimulation of cholinergic receptors and cadmium induced oxidative stress are capable of inducing elevated calcium and sodium ions concentration in the serum (Figure 2), an effect that is linked to the observed anxiety and altered motor function in these groups.

Nicotine-mediated cholinergic stimulation also involves the activation of neuropeptide Y and cAMP, which represents calcium dependent mechanisms involved in cholinergic hippocampal-mediated anxiety, epinephrine-mediated anxiety, and cholinergic neuromuscular stimulation [59–62]. Similarly, cadmium inhibits* cytochrome C* and* a3* in the mitochondria leading to ROS-dependent calcium release, anxiety, and synaptic excitotoxicity [63, 64]. Other studies have shown that cadmium affects the level of monoamine in the hypothalamus and hippocampus without a corresponding change in norepinephrine [63]. Interestingly, an increase in anxiety index was observed in the cadmium treated animals, similar to our findings. However, the mechanism of nicotine-induced bioaccumulation of cadmium and its relationship with the anxiety linked behavioural changes remains elusive. Based on the outcome of this study and previous studies, we hypothesise that cadmium-mediated synaptic inhibition, monoaminergic alterations, and induced calcium-shift are important in anxiety linked behaviour and decline in motor functions. As a result it complements the neural and behavioural changes attributed to nicotine in long term tobacco use.

## 5. Conclusion

Based on the outcome of this study, we infer that the motor and anxiety related behavioural changes observed in prolonged tobacco use in adult male mice are a result of the synergistic effect of nicotine-cadmium on synaptic plasticity, neurotransmitter function, and brain reward system. Also, nicotine and cadmium increased serum Ca^2+^ ions and caused no significant change in K^+^ ion concentration. However, combined nicotine-cadmium showed that nicotine facilitates bioaccumulation of cadmium in serum, resulting in a decrease in motor function and increased anxiety when compared with the nicotine and cadmium treatment groups. In addition, nicotine induced increase in weight during the acute and chronic phases while cadmium and cadmium-nicotine treatment caused a decrease in weight after the chronic phase. Ultimately, we conclude that nicotine and cadmium interaction in tobacco use is time-dependent and affects metabolism, electrolytes, and neurotransmitter functions that translate into the observed increased anxiety, decreased weight and altered motor function after chronic treatment.

## Supplementary Material

Table S1: Treatment and Animals grouping. Group A received 1.5mg/Kg cadmium chloride; Group B was treated with nicotine (0.4mg/Kg); Group C received cadmium and nicotine (1.5mg/Kg and 0.4mg/Kg respectively) while Group D received normal saline (control). The treatment was done in two phases; an acute phase (Day 1-7) and the chronic phase (Day 7-21).
