# Supplementary material for: Nicotine-Cadmium Interaction Alters Exploratory Motor Function and Increased Anxiety in Adult Male Mice
Source: J Neurodegener Dis. 2014 Nov 12;2014:359436. doi: 10.1155/2014/359436 (PMC4437340; doi:10.1155/2014/359436)
Supplement: Supplementary file 1 — Table S1: Treatment and Animals grouping. Group A received 1.5mg/Kg cadmium chloride; Group B was treated with nicotine (0.4mg/Kg); Group C received cadmium and nicotine (1.5mg/Kg and 0.4mg/Kg respectively) while Group D received normal saline (control). The treatment was done in two phases; an acute phase (Day 1-7) and the chronic phase (Day 7-21). [file 359436.f1.pdf]

Table S1: Animal grouping and treatment

Groups	Supplemental materials	Dose	Route of administration	Period
**Group(A) – Cd**	Cadmium chloride	1.5 mg/kg bw	Intraperitoneally (i.p)	7days (acute phase) for treatment 1 and another 7days(Chronic phase) for treatment 2
**Group(B) - Ni**	Nicotine	0.4 mg/kg bw	Sub-cutaneously (SC)	
**Group(C) – Cd + Ni**	Cadmium chloride and Nicotine	1.5 mg/kg bw + 0.4 mg/kg bw	i.p and SC, respectively	
**Group(D) - Control**	Normal saline	10 ml/kg bw	Intraperitoneally (i.p)	
